# Exploring COVID-19 Vaccine Intentions, Perceptions, and Uptake Among the Saudi Population

**DOI:** 10.7759/cureus.63404

**Published:** 2024-06-28

**Authors:** Ahmed A Bahri, Mohammad A Jareebi, Majed A Ryani, Jamaludeen A Othman, Mostafa Mohrag, Eyas A Akish, Mohammed O Shami, Hanan A Alessa, Jalal Y Madkhali, Khalid Bakri, Abdulaziz Hakami, Hamad A Otayn, Ahmad A Shok

**Affiliations:** 1 Department of Community and Family Medicine, Jazan University, Jazan, SAU; 2 Department of Community and Family Medicine, Faculty of Medicine, Jazan University, Jazan, SAU; 3 Department of Anesthesia and Critical Care, Jazan University Hospital, Jazan, SAU; 4 Department of Internal Medicine, Jazan University, Jazan, SAU; 5 Department of Medicine, Jazan University, Jazan, SAU; 6 College of Medicine, Jazan University, Jazan, SAU; 7 Department of Family Medicine, Health Affairs in Jazan Region, Jazan, SAU; 8 Department of Medicine and Surgery, Jazan University, Jazan, SAU; 9 Faculty of Medicine, Jazan University, Jazan, SAU; 10 Department of Pediatric Medicine, Health Affairs in Jazan Region, Jazan, SAU

**Keywords:** saudi arabia, promoting vaccination, vaccination acceptance, theory of planned behavior (tpb), health belief model (hbm)

## Abstract

Background

The widespread hesitancy, delays in acceptance, or outright refusal to receive vaccinations, even when readily available, present a notable global challenge. This is particularly pertinent in the context of COVID-19 vaccine hesitancy, where research shows considerable variability in hesitancy rates worldwide. The primary aim of this study is to investigate COVID-19 vaccine intentions among the Saudi population using behavioral theories as a framework.

Methodology

A descriptive cross-sectional study was conducted in the Kingdom of Saudi Arabia from January 2022 to December 2022. An online self-administered survey was distributed via social media to reach the targeted participants. Both men and women aged >18 years were included, while non-Saudi individuals and people who are less than 18 years old were excluded. A total of 1,139 participants were included.

Results

The study found that about 96% of respondents were willing to receive a COVID-19 vaccination. Analyzing sociodemographic and COVID-19-related factors with vaccine intention showed that those previously diagnosed with COVID-19 were less likely to refuse vaccination (*P* = 0.015). Univariate analyses revealed significant differences in the health belief model (HBM) and theory of planned behavior (TPB) dimensions between willing and unwilling participants. Willing individuals perceived greater infection severity (*P* = 0.543), higher vaccination benefits (*P* < 0.01), fewer barriers (*P* < 0.01), more cues to action (*P* < 0.01), and lower infection prevention self-efficacy (*P* < 0.01) compared to the unwilling group. TPB dimensions also differed, with willing individuals having more favorable vaccine attitudes (*P* < 0.01) and stronger vaccination social norms (*P* < 0.01). A multivariate logistic regression indicated that having COVID-19 increased vaccine willingness likelihood (odds ratio [OR] = 2.11, 95% confidence interval [CI] 1.14-3.91). Perceived barriers (OR = 0.61, 95% CI 0.44-0.85), self-efficacy (OR = 1.96, 95% CI 1.16-3.32) from HBM, and favorable vaccine attitudes (OR = 1.55, 95% CI 1.02-2.35) from TPB were significant predictors of vaccine willingness.

Conclusions

The TPB highlighted the importance of attitudes and perceived norms in vaccination acceptance, suggesting their value in vaccination promotion strategies. However, further research, including prospective and interventional studies drawing from a wider array of psychological theories, is needed to develop effective interventions for promoting vaccination.

## Introduction

In late 2019, COVID-19 emerged in Wuhan, China, becoming a pandemic on March 11, 2020 [1,2]. By January 2021, The virus had spread to more than 90 million individuals globally and led to approximately two million fatalities [3]. In Saudi Arabia, the first case was reported on March 2, 2020, with over 350,000 cases and 6,000 deaths by January 2021 [3,4]. Makkah Province had the highest COVID-19 case count, while Asir Province had the lowest. The Kingdom took strict measures like lockdowns, quarantine, travel restrictions, and canceling the hajj to control the virus spread of the virus [5]. This severely impacted the economy and social fabric of the Kingdom. Given the absence of a viable treatment, worldwide endeavors were focused on developing a COVID-19 vaccine that was both safe and effective [6,7].

Five COVID-19 vaccines reached Phase 3 trials, but only BNT162b2 and ChAdOx1 nCoV-19 efficacy and safety data were published. Saudi Arabia began vaccinating with BNT162b2 on December 17 [8,9]. A 67% vaccination rate among the 34 million population is crucial for herd immunity [10]. Challenges, including vaccine access, logistics, and public hesitancy, are influencing the success of the vaccination campaign [11].

The widespread hesitation, delays in acceptance, or outright refusal to receive vaccinations, especially when they are readily available, pose a significant global challenge [12]. Vaccine hesitancy presents a significant public health hurdle in Saudi Arabia, as evidenced by prior studies focusing on parental reluctance to vaccinate their children and hesitancy surrounding influenza vaccination [12]. Regarding COVID-19 vaccine hesitancy, research indicates significant variation in hesitancy rates globally. Reports indicate that Jordan has the highest percentage of COVID-19 vaccine hesitancy [13]. A specific study assessing vaccine hesitancy in selected cities in Saudi Arabia found that only 64.7% expressed intentions to receive a hypothetical vaccine [14]. To the best of our knowledge, there have been no studies investigating vaccine acceptance in the Kingdom after the COVID-19 vaccination campaign commenced. It's worth noting that reports on vaccination-related side effects and ongoing media discussions regarding the vaccine's efficacy and safety could potentially influence vaccine acceptance [15]. The health belief model (HBM) is a popular framework for evaluating vaccination beliefs and intentions. It has been widely utilized during epidemics to gauge people's attitudes toward diseases and vaccination [16].

The theory of planned behavior (TPB) is typically built upon three key predictors of behavioral intention: attitude toward a behavior, subjective norm, and perceived behavioral control. According to TPB, intentions are shaped by an individual's personal attitudes, perceptions of the act, and self-efficacy. Behavioral intention serves as a mediator between these three predictors and the actual behavior targeted. Due to the novel nature of preventive measures for COVID-19 in Saudi Arabia, these measures may be affected by surrounding people to determine the acceptability of engaging in the intended behavior in public [17]. According to our best knowledge, we found a limited paper interest study that dealt with this topic in the Kingdom of Saudi Arabia. The main objective of this study is to study the COVID-19 vaccine intentions in the Saudi population by application of behavioral theories.

## Materials and methods

A descriptive cross-sectional study was conducted in the Kingdom of Saudi Arabia from January 2022 to December 2022. An online self-administered survey was distributed via social media to reach the targeted participants. A convenience sampling technique was used to collect data from participants who were over 18 years old, including both men and women. Non-Saudi individuals and those under 18 years old were excluded from the study. The study was approved by the Standing Committee for Scientific Research, Jazan University (approval number REC-43/04/060, November 17, 2021).

The Raosoft sample size calculator (Raosoft Inc., Seattle, WA) was used to determine the sample size by considering the total population of 35,013,414 according to the General Authority for Statistics in the Kingdom of Saudi Arabia. We set a 95% confidence interval (CI), a 5% margin of error, and a 50% response distribution, which yielded a minimum sample size of 377.

However, to mitigate sampling bias in our methodology, given that this study relied on an online questionnaire distributed via social media, we expanded the sample size to encompass 1,139 participants. These participants were recruited through an online survey distributed via email and various social media platforms to ensure a more comprehensive and diverse representation.

Data collection

An online survey was carried out utilizing a Google form questionnaire among the Saudi population [9]. The questionnaire covered demographic information like age, gender, education level, COVID-19 illness history, and vaccination status. The second part of the survey delved into intentions and the stage of adopting preventive measures. This section was evaluated using a five-item dichotomous scale (yes/no) regarding current preventive behaviors. In a pilot study, test-retest reliability measures were performed to assess the stability of the instrument, yielding a reliability score of 0.80.

The third section focused on COVID-19 illness and vaccination, designed around the HBM to assess perceptions regarding vaccinations. It encompassed six domains of the model: perceived susceptibility, the severity of the illness, perceived benefits and barriers of vaccination, cues to action, and self-efficacy. Participants were asked to rate their agreement or disagreement with statements related to these dimensions using a Likert scale. The scale ranged from 1 = strongly disagree to 5 = strongly agree. A scale score was then computed for each construct by averaging the scores of all the relevant items. The scores for each item were further averaged to determine the scores for each independent category of the HBM and TPB. A higher scale score indicated a higher level of the corresponding factor.

The questionnaire utilized in this study was developed based on the HBM and TPB by a team consisting of epidemiologists, psychologists, and clinicians. Measures related to health beliefs and planned behavior were adapted from a previous study that employed the HBM and TPB models. The survey questions underwent rigorous testing for content validity and internal reliability. Content validity was assessed by experts in public health and preventive medicine, and based on their feedback and recommendations, adjustments were made to improve the survey tool.

Furthermore, the internal consistency of the HBM and TPB constructs was assessed using Cronbach's alpha coefficients. The Cronbach's alpha for the HBM constructs indicated a good internal consistency (Cronbach's α = 0.77), while the TPB constructs showed acceptable internal reliability (Cronbach's α = 0.60). Based on feedback received during the pilot study, appropriate revisions were made to enhance the questionnaire's effectiveness and reliability [18-20].

Data analysis

After collecting the data, a manual verification process was conducted followed by coding within an Excel spreadsheet. Subsequently, all the data were entered into IBM SPSS Statistics for Windows, Version 26.0 (IBM Corp., Armonk, NY). Descriptive statistics were computed for study variables, such as frequency and percentage for qualitative variables, and mean and standard deviation for quantitative variables. Tests of significance, including chi-square and t-tests, were applied as deemed appropriate for the analysis. A significance level of *P* < 0.05 was established to indicate statistical significance.

## Results

The sociodemographic and COVID-19-related characteristics are described in Table 1. Most participants were male (681, 59.8%), with a mean age of 31.22 ± 10.71 years, and holders of secondary school or less (1,017, 89.4%). A total of 318 (27.9%) had been tested positive for COVID-19.

**Table 1:** Distribution of study respondents by their intention to get the COVID-19 vaccination (n = 1,139). *P*-values were calculated using the chi-square test for categorical variables and the Kruskal-Wallis test for continuous variables. SAR, Saudi Riyals; SD, standard deviation

			COVID-19 vaccine intention			
Sociodemographic characteristics		Total (*n* = 1139) (%)	Willing (*n* = 1,095) (%)	Unwilling (*n* = 44) (%)	*χ*2	*P*-value
Gender	Male	681 (59.7)	658 (60.1)	23 (52.3)	1.076	0.347
	Female	458 (40.2)	437 (39.9)	21 (47.7)		
Age, mean (SD) (years)		31.22 (10.71)	31.17 (10.66)	32.44 (12.11)	0.438	0.508
Education	Secondary school or lower	1,017 (89.4)	981 (89.7)	36 (81.8)	3.086	0.214
	Bachelor	39 (3.4)	37 (3.4)	2 (4.5)		
	Higher studies	82 (7.2)	76 (6.9)	6 (13.6)		
Monthly income (SAR)	0-4,999	442 (38.8)	442 (38.5)	20 (45.5)	0.907	0.824
	5,000−9,999	245 (21.5)	236 (21.6)	9 (20.5)		
	10,000−15,000	213 (18.7)	206 (18.8)	7 (15.9)		
	>15,000	239 (21.0)	231 (21.1)	8 (18.2)		
COVID-19 status						
Tested positive for COVID-19	Yes	318 (27.9)	398 (27.2)	20 (45.5)	6.993	0.015
	No	821 (72.1)	797 (72.8)	24 (54.5)		

The proportion of respondents who were willing to take a COVID-19 vaccination was around 96% (1,095). A comparison between sociodemographic and COVID-19-related variables by vaccine intention indicated that individuals who had been diagnosed with COVID-19 (*P* = 0.015) were less likely to refuse a COVID-19 vaccination (Table 1).

A series of univariate analyses were run to compare different HBM and TPB dimensions regarding willingness to receive a COVID-19 vaccination (Table 2). All HBM dimensions, except perceived susceptibility (*P* = 0.287), showed differences between willing and unwilling participants. Compared to individuals who were unwilling to receive a COVID-19 vaccination, willing individuals perceived higher severity of the infection severity (*P* = 0.543), perceived greater benefits of vaccination (*P* < 0.01), had less barriers to vaccination (*P* < 0.01), had more cues to action (*P* < 0.01), and perceived less self-efficacy concerning prevention of infection (*P* < 0.01). Between-group differences were also observed for all TPB dimensions except perceived behavioral control (*P* = 0.543). Compared to the unwilling group, individuals who were willing to take a COVID-19 vaccination had more favorable attitudes toward the COVID-19 vaccine (*P* < 0.01) and better social norms regarding COVID-19 vaccination (*P* < 0.01).

**Table 2:** Univariate analysis of HBM and TPB dimensions regarding willingness to receive the COVID-19 vaccine (n = 1,139). A Kruskal–Wallis test. ^*^P < 0.05. ^**^P < 0.001. HBM, health behavior model; TPB, theory of planned behavior

Sociodemographic characteristics		COVID-19 vaccine intention			
	Total, mean (SD)	Willing, mean (SD)	Unwilling, mean (SD)	*χ*2	*P*-value
HBM					
Perceived susceptibility	3.10 (0.90)	3.11 (0.88)	2.95 (1.21)	1.133	0.287
Perceived severity	3.17 (1.02)	3.19 (0.99)	2.74 (1.47)	4.341	0.037^*^
Perceived barriers	2.96 (1.14)	2.93 (1.12)	3.58 (1.26)	13.943	0.000^**^
Perceived benefits	3.50 (1.04)	3.54 (1.00)	2.59 (1.46)	18.098	0.000^**^
Perceived self-efficacy (reverse coded)	3.66 (0.83)	3.68 (0.81)	2.97 (1.10)	20.080	0.000^**^
Cues to action	3.81 (0.99)	3.84 (0.95)	2.93 (1.54)	13.465	0.000^**^
TPB					
Attitude	3.96 (1.16)	4.01 (1.11)	2.70 (1.70)	24.881	0.000^**^
Social norms	3.85 (1.02)	3.88 (0.99)	3.06 (1.46)	12.735	0.000^**^
Perceived behavioral control	3.86 (1.30)	3.87 (1.28)	3.76 (1.69)	0.370	0.543

A hierarchical multivariate logistic regression was run and summarized in Table 3. According to the first model, which included only sociodemographic and COVID-19-related variables, individuals who had been diagnosed with COVID-19 at some points were more likely to be willing to take a COVID-19 vaccination (odds ratio [OR] = 2.11, 95% CI 1.14-3.91). According to the second model, which included HBM dimensions, two dimensions predicted the willingness to take the vaccine (*P* < 0.05). Intention to take a COVID-19 vaccination was significantly predicted by perceived barriers (OR = 0.61, 95% CI 0.44-0.85) and self-efficacy (OR = 1.96, 95% CI 1.16-3.32). According to the third model, which introduced TPB dimensions, having favorable attitudes toward COVID-19 vaccination was associated with intention to get vaccinated (OR = 1.55, 95% CI 1.02-2.35).

**Table 3:** Hierarchical multivariate logistic regression analysis of COVID-19 vaccination intention (n = 1,139). ^*^P < 0.05. HBM health behavior model; TPB, theory of planned behavior

	Odds ratio (95% confidence interval)		
Predictors	Model 1	Model 2	Model 3
COVID-19-related factors			
Tested positive for COVID-19			
No	Ref.	Ref.	Ref.
Yes	2.11 (1.14-3.91)^*^	1.87 (0.97-3.61)	1.82 (0.94-3.52)
HBM			
Perceived severity		1.34 (0.96-1.87)	1.28 (0.91-1.79)
Perceived barriers		0.61 (0.44-.85)^*^	0.67 (0.48-0.94)^*^
Perceived benefits		1.13 (0.71-1.78)	1.03 (0.65-1.65)
Perceived self-efficacy (reverse coded)		1.96 (1.16-3.32)^*^	1.85 (1.09-3.15)^*^
Cues to action		1.07 (0.68-1.68)	0.82 (0.48-1.41)^*^
TPB			
Attitude			1.55 (1.02-2.35)^*^
Social norms			0.96 (0.70-1.34)
Model summary			
Cox and Snell pseudo R^2^	0.01	0.05	0.05
Nagelkerke pseudo R^2^	0.02	0.17	0.18

## Discussion

Vaccination emerges as an effective preventive strategy to mitigate the global impact of the COVID-19 pandemic [12]. Despite the implementation of various preventive measures and extensive efforts to vaccinate a significant portion of their populations, the reluctance or refusal to receive vaccination, even when readily available, remains a major global issue. Vaccine hesitancy is a recurring concern, especially with the introduction of new vaccines [21]. The rapid development of COVID-19 vaccines during the pandemic may further exacerbate this hesitancy [5]. To delve into the hesitancy and intentions regarding vaccination, we conducted a theory-based, cross-sectional study aimed at identifying the intentions, perceptions, and uptake of COVID-19 vaccines among the Saudi population.

The present study investigates the intentions of the Saudi population regarding COVID-19 vaccination and investigates predictive constructs related to vaccination behavior using behavioral theories. It employed a combination of the HBM and the TPB, which are instrumental in understanding the acceptance and adoption of preventive interventions. The findings revealed that 96% of the respondents either received the COVID-19 vaccine or expressed willingness to do so, indicating a high percentage of individuals willing to receive the vaccine.

Most of the HBM constructs, including perceived severity, barriers, benefits, cues to action, and self-efficacy, were identified as reliable predictors of vaccination acceptance. However, the exception was perceived susceptibility, which did not significantly predict vaccination acceptance behavior. Perceived benefits reflect an individual's perception of the positive outcomes resulting from an action, while perceived barriers represent one's views on obstacles to performing a specific action. Both perceptions were linked to the intention to receive a vaccine. Self-efficacy denotes an individual's belief in their ability to carry out a behavior, and this construct was a strong predictor of vaccination acceptance (*P* < 0.001). Cues to action prompt the decision-making process to accept or engage in a recommended health action, and the association between this construct and vaccination acceptance was statistically significant (*P* < 0.001). However, further studies indicated that the only HBM construct not predictive of behavior is *perceived severity* [22,23].

The majority of the participants indicated positive attitudes toward COVID-19 vaccines. These attitudes have been proven to predict intention, as indicated by TPB [24]. This finding is consistent with other studies in the United Kingdom [18], the United States [19], and Pakistan [20]. In this current study’s findings, the perceived norm was strongly associated with receiving a vaccine. This finding is compatible with different articles that studied intention through TPB [19]. The last construct of TPB is perceived behavioral control. The current study showed that the association between perceived behavioral control with intentions to vaccinate was not statistically significant and a regression analysis showed that they did not predict such intentions. This finding is consistent with other studies on COVID-19 vaccines, which have shown that perceived control was not a significant predictor of vaccination intention. This has been noted in several studies, including those from China [25] and the United States [19]. On the other hand, Ullah et al. [20] discovered a significant association between perceived control and the intention to get vaccinated.

Overall, this study offers valuable insights into how psychological models, specifically the HBM and the TPB, can influence vaccination uptake among individuals. However, several limitations should be acknowledged before using these findings to guide intervention development.

First, the study relied on cross-sectional data, making it challenging to interpret the associations identified due to the nature and design of the study. Second, the convenience sampling method and recruitment strategies employed may impact the generalizability of the results. Despite these limitations, the study provides a descriptive and exploratory analysis of COVID-19 vaccine acceptance and intentions based on the constructs of HBM and TPB.

## Conclusions

The HBM emerged as the most frequently utilized model in this study, demonstrating support for its application, especially in domains such as perceived benefits, perceived risk, perceived severity, and cues to action. The TPB elucidated the significance of attitudes and perceived norms among the target population regarding vaccination acceptance. These constructs could serve as valuable targets in vaccination promotion strategies. However, this study underscores the necessity for further research in the field of vaccination behavior, particularly prospective and interventional studies of high methodological quality that draw from a broader range of psychological theories. Additional evidence would be beneficial in shaping the development of effective interventions for promoting vaccination.
